# Tonsil-derived mesenchymal stem cells enhance allogeneic bone marrow engraftment via collagen IV degradation

**DOI:** 10.1186/s13287-021-02414-6

**Published:** 2021-06-05

**Authors:** Hyun-Ji Lee, Yu-Hee Kim, Da-Won Choi, Kyung-Ah Cho, Joo-Won Park, Sang-Jin Shin, Inho Jo, So-Youn Woo, Kyung-Ha Ryu

**Affiliations:** 1 Department of Microbiology, College of Medicine, Ewha Womans University, Gangseo-Gu, Seoul, Republic of Korea; 2 Graduate Program in System Health Science and Engineering, Ewha Womans University, Seodaemun-gu, Seoul, Republic of Korea; 3 Department of Biochemistry, Ewha Womans University, Gangseo-Gu, Seoul, Republic of Korea; 4 Department of Orthopaedic Surgery, Ewha Womans University, Gangseo-Gu, Seoul, Republic of Korea; 5 Department of Molecular Medicine, Ewha Womans University, Gangseo-Gu, Seoul, Republic of Korea; 6 Department of Pediatrics, College of Medicine, Ewha Womans University, Gangseo-Gu, Seoul, 07804 Republic of Korea

**Keywords:** Tonsil-derived mesenchymal stem cells, Metalloproteinase-3, Allogeneic bone marrow transplantation, Engraftment, Type IV collagen

## Abstract

**Background:**

Co-transplantation of bone marrow cells (BMCs) and mesenchymal stem cells (MSCs) is used as a strategy to improve the outcomes of bone marrow transplantation. Tonsil-derived MSCs (TMSCs) are a promising source of MSCs for co-transplantation. Previous studies have shown that TMSCs or conditioned media from TMSCs (TMSC-CM) enhance BMC engraftment. However, the factors in TMSCs that promote better engraftment have not yet been identified.

**Methods:**

Mice were subjected to a myeloablative regimen of busulfan and cyclophosphamide, and the mRNA expression in the bone marrow was analyzed using an extracellular matrix (ECM) and adhesion molecule-targeted polymerase chain reaction (PCR) array. Nano-liquid chromatography with tandem mass spectrometry, real-time quantitative PCR, western blots, and enzyme-linked immunosorbent assays were used to compare the expression levels of metalloproteinase 3 (MMP3) in MSCs derived from various tissues, including the tonsils, bone marrow, adipose tissue, and umbilical cord. Recipient mice were conditioned with busulfan and cyclophosphamide, and BMCs, either as a sole population or with control or MMP3-knockdown TMSCs, were co-transplanted into these mice. The effects of TMSC-expressed MMP3 were investigated. Additionally, Enzchek collagenase and Transwell migration assays were used to confirm that the collagenase activity of TMSC-expressed MMP3 enhanced BMC migration.

**Results:**

Mice subjected to the myeloablative regimen exhibited increased mRNA expression of collagen type IV alpha 1/2 (*Col4a1* and *Col4a2*). Among the various extracellular matrix-modulating proteins secreted by TMSCs, MMP3 was expressed at higher levels in TMSCs than in other MSCs. Mice co-transplanted with BMCs and control TMSCs exhibited a higher survival rate, weight recovery, and bone marrow cellularity compared with mice co-transplanted with BMCs and MMP3-knockdown TMSCs. Control TMSC-CM possessed higher collagenase activity against collagen IV than MMP3-knockdown TMSC-CM. TMSC-CM also accelerated BMC migration by degrading collagen IV in vitro.

**Conclusions:**

Collectively, these results indicate that TMSCs enhance BMC engraftment by the secretion of MMP3 for the modulation of the bone marrow extracellular matrix.

## Background

Hematopoietic stem cell transplantation (HSCT) is the most potent therapy for patients with hematological malignancies. However, conventional HSCT still results in high mortality, early and late stages after HSCT [1, 2], with 50–60% of such deaths occurring due to graft failure [3]. Co-transplantation of mesenchymal stem cells (MSCs) is among the various strategies developed to improve the outcomes of HSCT [4–6]. Clinical and preclinical studies have demonstrated effects of MSC co-transplantation on enhancing HSCT efficacy [7, 8].

Human tonsil-derived MSCs (TMSCs), isolated from human palatine tonsil tissues [9], have been suggested as an attractive source of MSCs because of their high proliferation rate, low immunogenicity, and ease of isolation using noninvasive methods [10, 11]. Our research group has been investigating therapeutic effects of TMSCs and their mechanisms of action. By performing transcriptomic and proteomic analyses, we screened molecules with higher expression levels in TMSCs compared to bone marrow (BM) or adipose tissue (AD) MSCs [12, 13]. Furthermore, we have reported immune modulating, anti-fibrotic, and insulin sensitizing effects of TMSCs via secreting PD-L1, IL-1Ra, and IGFBP5, respectively [13–15]. TMSCs have also been expected to enhance bone marrow transplantation (BMT) outcomes. Previous studies have shown that the co-transplantation of bone marrow cells (BMCs) and TMSCs enhances peripheral mononuclear cell production [16] and immune system recovery [17]. Additionally, conditioned media from TMSCs (TMSC-CM) attenuate acute graft-versus-host disease by secretion of tumor necrosis factor-stimulated gene 6 [18] and improve BMC migration via recovery of the endothelium by secreting pleiotrophin [19]. However, the factors expressed in co-transplanted TMSCs that enhance bone marrow engraftment have not yet been identified.

Unlike other types of organ transplantation, BMT involves infusion of cells via peripheral vein since direct transplantation into the bone marrow would results in fat and air embolism. Transplanted BMCs undergo the processes of homing and engraftment over a month. During this period, the patient experiences pancytopenia and is under a high risk of bleeding and infection. Therefore, development of therapeutic strategies that can shorten the process of engraftment is in demand. Advantages of MSC co-transplantation in hematopoietic recovery have been reported in clinical studies [20, 21]. However, the underlying mechanisms of action have not been fully understood.

The engraftment of BMCs is regulated by the bone marrow niche, which is composed of chemotactic factors and extracellular matrix (ECM) proteins [22]. Major chemotactic factors such as α-chemokine stromal-derived factor 1 (SDF-1), sphingosine-1-phosphate, and ceramide-1-phosphate retain and regulate both the migration and homing of HSCs [23]. The ECM contains proteoglycans, fibrous proteins, glycosaminoglycans, and matricellular proteins, which support various myeloid cell activities [24, 25]. When irradiated mice are deficient in laminin, a fibrous protein of the bone marrow, BMC engraftment is significantly less efficient, as seen in competitive BMC transplantation (BMT) assays [26]. In order to develop a novel strategy to enhance the bone marrow engraftment, we focused on the modulation of ECM proteins by MSCs. To offer supporting evidence, we first investigated the changes in bone marrow ECM constituents following preconditioning with high doses of cytotoxic drugs. Next, we selected an ECM-modulating molecule secreted from TMSCs by analyzing proteomics data and examined the role of the candidate molecule using a loss-of-function study.

Some matrix metalloproteinases (MMPs) such as MMP2, MMP9, and MT1-MMP (MMP14) degrade the ECM barrier, allowing the migration of hematopoietic stem/progenitor cells (HSPCs) across the sinusoid endothelium during cellular chemotaxis in response to CXCR4/SDF-1 [27]. MMP3, also known as stromelysin 1, is a member of the MMP family. MMP3 degrades ECM proteins, such as collagen types II, IV, and IX, and activates other MMPs, such as MMP1, 7, and 9. MMP3 has also been shown to facilitate cellular migration and invasion [28, 29], but whether it plays a role in engraftment has not yet been determined. Hence, in this study, we investigated whether TMSCs enhance BMC engraftment via the secretion of MMP3.

## Methods

### Mice

BALB/c and C57BL/6 mice were purchased from OrientBio (Sungnam, Korea). Mice were housed at 21Dis°C with 51–54% humidity in a pathogen-free environment under a 12-h light/dark cycle and were given full access to food and water. The Animal Care and Use Committee of Ewha Womans University College of Medicine approved all experimental procedures and protocols (Seoul, Korea; approval no. EUM20-014).

### Cells

Previously isolated and maintained TMSCs were cultured in DMEM low-glucose medium supplemented with 10% (v/v) fetal bovine serum (FBS; Welgene, Gyeongsan, Korea) [9, 30]. Bone marrow-derived MSCs (BMMSCs; PCS-500-012) were purchased from the ATCC (Manassas, VA, USA); adipose tissue-derived MSCs (ADMSCs; C-12977) and Wharton’s jelly-derived MSCs (WJMSCs; C-12971) were obtained from PromoCell (Heidelberg, Germany). MSCs were maintained as previously described [18]. To determine gene and protein expression levels, MSCs from passages 7 to 9 were used.

### siRNA transfection

TMSCs at a density of 3 × 10^5^/well were cultured in serum-containing DMEM in 6-well plates for 24-h and transfected with either 7.5 nM MMP3 siRNA (target sequence: 5′-TTGGCGCAAATCCCTCAGGAA-3′; Qiagen, Hildesheim, Germany) or non-silencing control siRNA (target sequence: 5′-TTGGCGCAAATCCCTCAGGAA-3′; Qiagen) for 72 h. Cells were incubated in serum-containing DMEM for additional 72 h.

### Myeloablative preconditioning

To evaluate the effects of preconditioning using a combination of busulfan and cyclophosphamide (Bu/Cy) on the ECM of the bone marrow, BALB/c mice were divided into two groups: normal and Bu/Cy (n = 15/group). The Bu/Cy group received 20 mg/kg/day Bu for 4 days (Sigma-Aldrich, St. Louis, MO, USA) and 100 mg/kg/day Cy for 2 consecutive days (Baxter Oncology, Westfalen, Germany). The mice were sacrificed after 48 h by isoflurane anesthesia (JW Pharma, Seoul, Korea) and cervical dislocation. Two femurs and two tibias per mouse were collected and used for further analysis.

### BMC preparation

For BMT and analyses using real-time quantitative PCR (RT-qPCR), flow cytometry, and migration assays, BMCs were prepared as follows: The isolated femurs or tibias were washed sequentially with DMEM, PBS, 70% ethanol, and PBS before immersion in serum-free DMEM. Bone ends were cut, and bone marrow was flushed from the medullary cavities with DMEM using a 5-mL syringe. The BMC suspensions were filtered using a 70-μm cell strainer (SPL Life Sciences, Pocheon, Korea) and centrifuged at 190 × *g* at room temperature for 5 min. To remove erythrocytes, the cell pellet was resuspended and incubated in lysis buffer containing ammonium, chloride, and potassium (ACK; 150 mM NH_4_Cl, 10 mM KHCO_3_, and 0.1 mM Na_2_ EDTA) at room temperature for 5 min. After diluting the suspension five-fold with PBS, BMCs were collected by centrifugation and used for further analysis.

### BMT

BMT was performed as previously described with a slight modification [17]. Recipient BALB/c mice received 25 mg/kg/day Bu for 4 days and 100 mg/kg/day Cy for 2 consecutive days (Bu/Cy) to induce bone marrow ablation [31]. After 48 h, mice were transplanted with 10^7^ BMCs isolated from C57BL/6 donor mice with or without 10^6^ TMSCs, transfected with control or MMP3 siRNA via lateral tail vein injection. We decided to co-transplant 10^6^ TMSCs, as it showed a significant increase in mouse survival (data not shown). For donor BMCs, C57BL/6 donor mice were sacrificed, and erythrocyte-deficient BMCs were prepared as a single-cell suspension. The cell pellets were resuspended in serum-free DMEM for injection. The mice were divided into five groups as follows: the first group was the control, which received neither Bu/Cy preconditioning nor BMT (Control); the second group received only Bu/Cy preconditioning but not BMT (BuCy); the third group received Bu/Cy preconditioning followed by BMT with 10^7^ donor BMCs (BMC); the fourth group received Bu/Cy preconditioning followed by BMT with 10^7^ donor BMCs and 10^6^ control siRNA-transfected TMSCs (BMC + TMSC); and the fifth group received Bu/Cy preconditioning followed by BMT with 10^7^ donor BMCs and 10^6^ MMP3 siRNA-transfected TMSCs (BMC + MMP3kdTMSC). A humane endpoint was established as the time when the animal’s body weight was the 75% of its starting weight. During the experimental period, mice at the humane endpoint were sacrificed immediately. The average weights of each group were recorded. Mice were sacrificed 10 days after transplantation.

### Hematoxylin and eosin (H&E) staining

To assess engraftment, bone marrow cellularity was examined by H&E staining, as previously described [19]. Paraffin-embedded sections were deparaffinized twice in xylene (JUNSEI Chemical, Tokyo, Japan) for 5 min each and serially hydrated in 100%, 95%, 90%, 80%, and 70% ethanol for 3 min each and distilled water for 10 min. Slides were washed twice with PBS for 5 min each, stained with hematoxylin (YD Diagnostics, Yongin, Korea) for 2 min, and immersed in ethanol containing 1% hydrogen chloride three times. After washing in tap water for 5 min, slides were stained in eosin (Sigma-Aldrich) for 1 min and washed in tap water again. For dehydration, slides were immersed in 70%, 80%, 90%, 95%, and 100% ethanol for 1 min each and in xylene for 3 min, followed by mounting using synthetic mountant (Thermo Fisher Scientific, Waltham, MA, USA). After mounting, slides were scanned using a bright field ScanScope slide scanner (Aperio SC2, Leica Biosystems, Buffalo Grove, IL, USA), and images were captured at either × 20 or × 200 magnification using Aperio ImageScope software, v12 (Leica Biosystems). The stained areas were identified using ImageJ software (https://imagej.nih.gov/ij/download.html). Bone marrow cellularity was defined as the ratio of the hematoxylin-stained area within the medullary cavity of the diaphysis, corresponding to the area occupied by BMCs, to the selected total area.

### Immunohistochemistry

For immunohistochemical analysis of collagen IV, femur tissues were deparaffinized by immersion in xylene twice for 5 min and hydrated serially by 95%, 90%, 85%, 80%, and 70% ethanol for 3 min each and distilled water for 10 min. For a heat mediated epitope retrieval, the slides were dipped in citrated buffer (10 mM citrate containing 0.05% Tween 20, pH 6.0) at 95 °C for 15 min and cooled down. Peroxidase blocking for 30 min at RT and protein blocking for 1 h at RT were performed using a Dako reagents (Agilent, Santa Clara, CA, USA) then specimens were incubated with anti-Collagen IV primary antibody (1:200, Abcam, Cambridge, MA) overnight at 4 °C. On the following day, slides were incubated with a Dako LSAB2 System-HRP for 30 min at RT then incubated with streptavidin conjugated to HRP for 30 min. The slides were developed with 3,3′-diaminobenzidine solution for 2 min and washed in running tap water then counterstained with hematoxylin for 60 s. After washing, slides were dehydrated and mounted. Collagen IV expression was and observed using a slide scanner.

### Flow cytometry

To determine MHC haplotype using flow cytometry, cells were stained with PE/Cyanine7-conjugated anti-mouse H-2^b^ (28-8-6, mouse IgG2a; Biolegend, San Diego, CA, USA) and Alexa flour 488-conjugated anti-mouse H-2^d^ (SF1-1.1, mouse IgG2a; Biolegend) antibodies. The expression was measured using NovoCyte flow cytometer (ACEA Biosciences, San Diego, CA, USA) and analyzed using NovoExpress software.

### Hematological analysis

Mouse blood samples were collected into EDTA-containing tubes (Golden Vac0, Hermosillo, Mexico) from the submandibular vein on day 21 post BM transplantation. Numbers of red blood cells (RBCs) and white blood cells (WBCs) were counted using Auto Hematology Analyzer (BC-2800Vet; Mindray, Shenzhen, China).

### RT-qPCR

To confirm the effects of Bu/Cy preconditioning on the ECM of mouse bone marrow, mRNA expression levels of mouse ECM and adhesion molecules in BMCs from the control and BuCy groups were determined using an RT2 profiler PCR array kit (PAMM-013ZA; Qiagen, Valencia, CA, USA; n = 3/group). Data were analyzed using the Excel sheet provided by the manufacturer (RT2 Profiler PCR Array Data Analysis Spreadsheet 1904; https://www.qiagen.com/ca/resources/resourcedetail?id=b3396407-ecb5-4656-ac5d-5ea7b83a397e&lang=en). *Gusb* and *β-actin* were selected as the reference genes. To verify the array results, mRNA expression levels of *Col4a1* and *Col4a2* were determined by RT-qPCR using custom oligos (Macrogen, Seoul, Korea). *Gusb* was used as the reference gene. The mRNA expression levels of MMP3, MMP1, CTSB, and PEPD in human TMSCs, BMMSCs, and ADMSCs were also determined by RT-qPCR using custom oligos (Macrogen). *GAPDH* was used as the reference gene. After the cycle threshold (Ct) value was obtained, relative mRNA expression levels were calculated using the following formula: 2 (Ct of the reference gene – Ct of the target gene). The fold change was calculated as the ratio of the relative expression level of the test group to that of the control group.

### Proteomics

The conditioned media of various MSCs were obtained, as previously reported with a few modifications [18]. Cells at 80% confluence were washed twice with PBS and incubated in serum-free DMEM for 48 h. The conditioned media were collected, filtered by centrifugation at 190 × *g* at room temperature for 5 min to remove suspended cells and concentrated using an Amicon Ultra Centrifugal Filter unit (molecular weight cut-off value of 3KDa; Merck, Darmstadt, Germany) by high-speed centrifugation at 5000 × *g* at 4 °C for 1 h. Nano-liquid chromatography coupled with tandem mass spectrometry (nano LC-MS/MS) analysis using the Mascot algorithm (Matrix Science, Boston, MA, USA) was performed, as previously reported [13].

### Western blot

To quantify MMP3 protein levels in MSC lysates, western blots were performed. After washing cells twice with ice-cold PBS, cells were incubated on ice for 15 min in protein lysis buffer [20 mM HEPES, 1% Triton X-100, 150 mM NaCl, 1 mM EDTA, 2 mM Na_3_VO4, 10 mM NaF, and protease inhibitor cocktail (P8340, Sigma-Aldrich)]. Cells were harvested using cell scrapers, and cell lysates were prepared by centrifugation at 15,520 × *g* for 15 min at 4 °C. The supernatant was collected, and total protein concentrations were determined by the bicinchoninic acid (BCA) assay using the BCA Protein Assay kit (Pierce, Thermo Fischer Scientific). Each cell lysate containing 15 μg total protein was electrophoresed through a 12% polyacrylamide gel. The gels were then transferred to polyvinylidene difluoride (PVDF) membranes (EMD Millipore, Merck). The membranes were blocked with 5% skim milk in Tris-buffered saline containing 0.1% Tween 20 (TBST; 50 mM Tris-HCl, pH 7.6, 150 mM NaCl, and 0.1% Tween-20) and incubated with anti-MMP3 mouse monoclonal antibody (sc-21732; 57 kDa; 1:500 ;Santa Cruz Biotechnology, Santa Cruz, CA, US) or anti-β-actin mouse monoclonal antibody (sc-47778; 43 kDa; 1:1,000; Santa Cruz Biotechnology) overnight at 4 °C. All primary antibodies were diluted with TBST containing 3% BSA. The PVDF membranes were washed three times in TBST for 10 min each and incubated with secondary goat anti-mouse IgG (H + L)-HRP antibody (#1706516; 1:3,000; Bio-Rad, Hercules, CA, USA) in TBST at room temperature for 1 h. The membranes were then washed three times in TBST for 10 min each and developed using an enhanced chemiluminescent solution (EZ-Western Lumi Femto; doGenBio, Seoul, Korea). Images were obtained using the ImageQuant LAS 3000 (GE Healthcare, Little Chalfont, UK). The pixel densities of the MMP3 bands were divided by the pixel densities of the corresponding β-actin bands for protein quantitation using UN-SCAN-IT-gel 6.1 software (Silk Scientific, Orem, UT, USA; https://www.silkscientific.com/gel-analysis.htm).

### ELISA

To quantify the MMP3 protein concentrations in MSC-CM, an enzyme-linked immunosorbent assay (ELISA) was conducted using a human MMP3 ELISA kit (RayBiotech, Norcross, GA, USA), according to the manufacturer’s instructions.

### Collagenase assay

The enzymatic activity of MMP3 against collagen IV was investigated using the EnzChek gelatinase/collagenase assay kit and fluorescently labeled DQ-type IV collagen (Thermo Fisher Scientific), according to the manufacturer’s instructions. CM were incubated with 5 μg fluorescently labeled collagen IV in a microplate at 37 °C in the dark. After 18 h, fluorescence was determined at 493 nm (excitation)/518 nm (emission) using an ELISA reader.

### Transwell migration assay

Collagen IV (at 10 μg/cm^2^; CAS 9007-34-5; C6745; Sigma-Aldrich) was coated on the membrane inserts in a 24-well Transwell plate (5-μM pore size; Costar, Corning, Corning, NY, USA). BMCs were isolated from normal healthy C57BL/6 mice and suspended at a density of 2 × 10^5^ cells/50 μL in DMEM. The cell suspensions were mixed with 50 μL TMSC-CM. Six hundred microliters of 100 ng/mL mouse SDF-1 (chm-324; ProSpec, Rehovot, Israel) were added to the receiving well, and 100 μL of the cell suspension was placed onto a membrane insert. Cells were incubated for 6 h in an CO_2_ incubator. The number of BMCs that migrated into the lower chamber was determined using 0.4% trypan blue staining (Gibco, Thermo Fisher Scientific) and flow cytometry.

### Statistical analysis

Data represent the mean ± standard error of the mean (S.E.M.). Statistical significance was determined using the Student’s *t* test for comparisons of two groups, one-way analysis of variance (ANOVA) was used for comparisons of more than three groups with one independent factor, and two-way ANOVA was used for multiple comparisons of more than three groups with two independent factors. Survival curves were plotted using Kaplan-Meier estimates. All analyses were performed using GraphPad Prism software, v8 (GraphPad Software, La Jolla, CA, USA). For all analyses, *P* values less than 0.05 were considered statistically significant.

## Results

### Bu/Cy preconditioning changes bone marrow cellularity and ECM-related gene expression

**Fig. 1:**
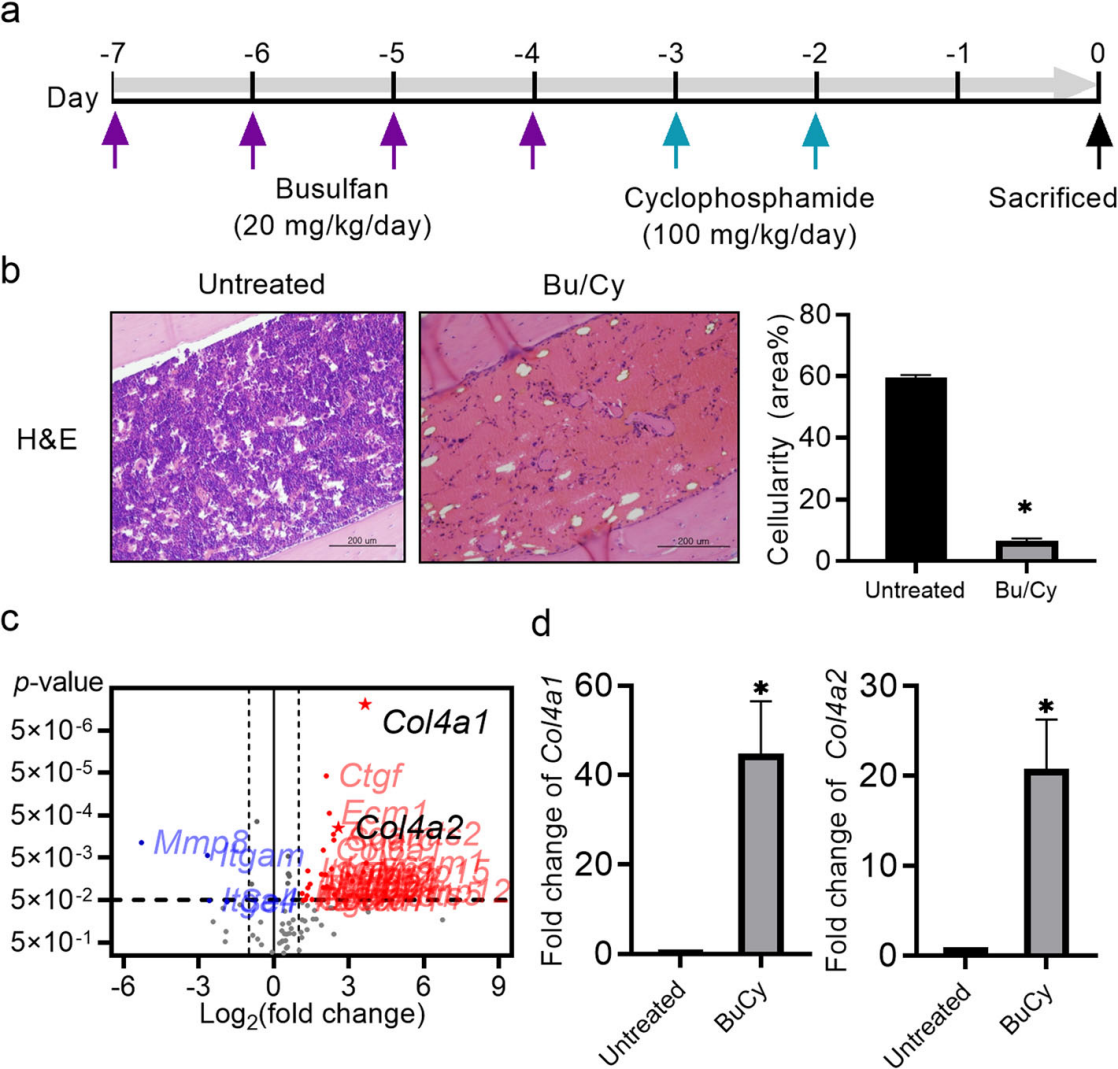
Bu/Cy preconditioning changes bone marrow cellularity and upregulates Col4a1 and Col4a2 expression in bone marrow. **a** Schematic representation of the experimental timeline. BALB/c mice were divided into two groups: untreated control and Bu/Cy (n = 5/group). The Bu/Cy group received 20 mg/kg/day Bu for 4 days and 100 mg/kg/day Cy for 2 consecutive days. After 48 h, mice were sacrificed and used for further analysis. **b** Slides of mouse femur sections were stained with hematoxylin and eosin (H&E). Cellularity was defined as the ratio of the hematoxylin-stained area within the medullary cavity of the diaphysis, corresponding to the area occupied by BMCs, to the selected total area. Scare bar 200 μm. **c** The RT2 profiler PCR array (for the analysis of mouse ECM and adhesion molecule mRNA expression) was used for real-time quantitative PCR (RT-qPCR) (n = 3/group). Data were analyzed using the Excel sheet provided by the manufacturer. Gusb and β-actin were selected as reference genes. **d** The mRNA expression levels of Col4a1 (left) and Col4a2 (right) were verified by RT-qPCR using custom oligos. The data represent the mean ± S.E.M. and were analyzed using the Student’s t test (*P < 0.05)

We hypothesized that Bu/Cy preconditioning regimen induces composition changes in the ECM of the bone marrow. To investigate this, preconditioned BALB/c mice were sacrificed after 48 h, and histological changes of bone marrow and mRNA expression changes of BMCs were analyzed (Fig. 1a). The Bu/Cy myeloablative regimen decreased bone marrow cellularity to 5%, drastically lower than the 60% bone marrow cellularity found in untreated control mice (Fig. 1b). The expression levels of 84 genes related to the ECM and adhesion molecules were analyzed using a pathway-targeted PCR array, and results revealed that Bu/Cy treatment altered 30 genes related to ECM and adhesion molecules in BMCs (Table 1). Among the ECM-related genes *Col4a1* and *Col4a2*, the subunits of collagen type IV (Col IV) were highly upregulated by 12.51- and 5.95-fold, respectively (Fig. 1c). The array results were confirmed using RT-qPCR, and data showed that Bu/Cy treatment significantly increased the expression levels of *Col4a1* and *Col4a2* (Fig. 1d).

**Table 1:** List of mouse ECM and adhesion molecule genes in the Bu/Cy group whose expression levels were changed more than two-fold compared to the normal group

Functional category	Gene symbol	Accession no.	Gene name	Fold change
Basement membrane constituents	*Col4a1*	NM_009931	Collagen type IV alpha 1	12.51
	*Col4a2*	NM_009932	Collagen type IV alpha 2	5.95
	*Sparc*	NM_009242	Secreted acidic cysteine rich glycoprotein	5.24
	*Lamc1*	NM_010683	Laminin, gamma 1	3.95
	*Timp2*	NM_011594	Tissue inhibitor of metalloproteinase 2	2.55
Collagens and ECM structural constituents	*Col6a1*	NM_009933	Collagen type VI alpha 1	3.92
	*Col1a1*	NM_007742	Collagen type I alpha 1	2.39
Other ECM molecules	*Tnc*	NM_011607	Tenascin C	7.91
	*Ecm1*	NM_007899	Extracellular matrix protein 1	4.67
	*Ctgf*	NM_010217	Connective tissue growth factor	2.10
ECM proteases	*Mmp12*	NM_008605	Matrix metallopeptidase 12	21.45
	*Mmp15*	NM_008609	Matrix metallopeptidase 15	11.89
	*Adamts5*	NM_011782	A disintegrin-like and metallopeptidase with thrombospondin type 1 motif, 5 (aggrecanase-2)	5.33
	*Adamts2*	NM_175643	A disintegrin-like and metallopeptidase with thrombospondin type 1 motif, 2	5.20
(ECM proteases)	*Mmp2*	NM_008610	Matrix metallopeptidase 2	4.20
	*Mmp14*	NM_008608	Matrix metallopeptidase 14	2.70
	*Adamts1*	NM_009621	A disintegrin-like and metallopeptidase with thrombospondin type 1 motif, 1	2.50
	*Mmp8*	NM_008611	Matrix metallopeptidase 8	− 38.97
Cell-ECM adhesion	*Itga3*	NM_013565	Integrin alpha 3	8.77
	*Itgax*	NM_021334	Integrin alpha X	2.89
	*Itga5*	NM_010577	Integrin alpha 5	2.57
	*Itgav*	NM_008402	Integrin alpha V	2.19
	*Itga4*	NM_010576	Integrin alpha 4	− 5.94
	*Itgam*	NM_008401	Integrin alpha M	− 6.29
Transmembrane receptors	*Vcam1*	NM_011693	Vascular cell adhesion molecule 1	12.94
	*Icam1*	NM_010493	Intercellular adhesion molecule 1	4.96
	*Cdh2*	NM_007664	Cadherin 2	4.36
	*Selp*	NM_011347	Selectin, platelet	3.74
	*Pecam1*	NM_008816	Platelet/endothelial cell adhesion molecule 1	2.26
	*Sell*	NM_011346	Selectin, lymphocyte	− 3.65

### MMP3 is secreted from TMSCs

**Fig. 2:**
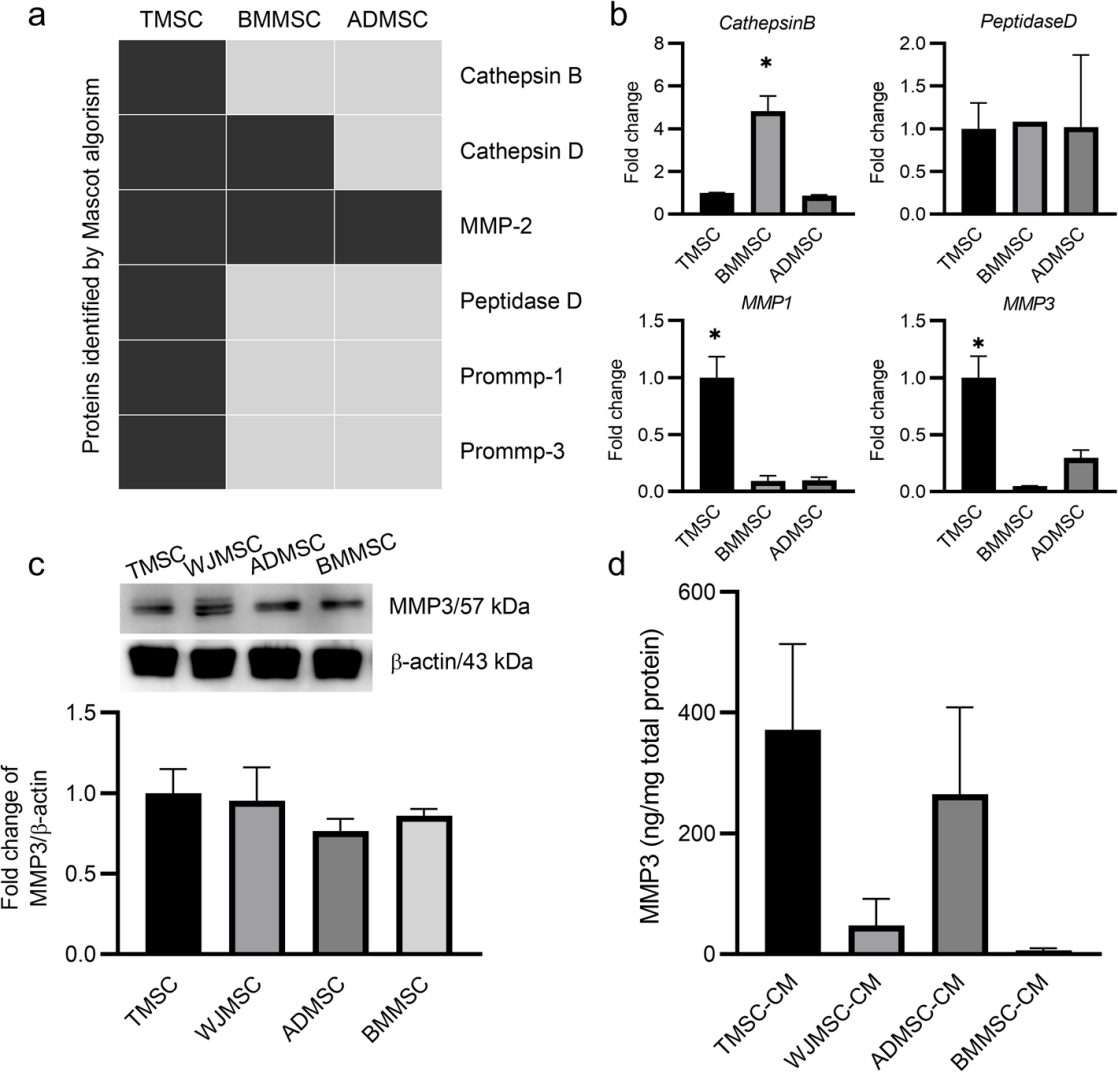
MMP3 expression and secretion is increased in TMSCs compared to other MSCs. **a** Nano-liquid chromatography with tandem mass spectrometry analysis was performed using TMSC-CM, BMMSC-CM, and ADMSC-CM. Peptides of ECM-modulating enzymes are listed. Detected peptides are depicted in black. **b** The mRNA expression levels of MMP3, MMP1, CTSB, and PEPD in human TMSCs, BMMSCs, and ADMSCs were determined using real-time quantitative PCR (RT-qPCR) with custom oligos. GAPDH was used as the reference gene. **c** MMP3 protein expression levels in MSC lysates were determined by quantitative western blot analysis by dividing the pixel densities of the MMP3 bands by the pixel densities of the corresponding β-actin bands. **d** MMP3 protein concentrations in MSC-CM were quantified using a human MMP3 ELISA kit. The data represent the mean ± S.E.M. and were analyzed using one-way ANOVA (*P < 0.05)

Next, we screened ECM-modulating factors secreted from MSCs. Proteomic analysis was performed using the CM of TMSCs, BMMSCs, and ADMSCs by nano LC-MS/MS and Mascot algorithm software. Among ECM-modulating proteins, cathepsin B, peptidase D, prommp-1, and prommp-3 peptides were detected only in TMSC-CM (Fig. 2a). The results were confirmed using RT-qPCR, and the mRNA expression levels of *MMP1* and *MMP3* were consistent with the proteomics data (Fig. 2b). We further investigated the role of MMP3 because MMP3, but not MMP1, has known collagenase activity against collagen IV [28], which is shown to be upregulated after preconditioning. MMP3 protein expression levels were analyzed using cell lysates and CM of TMSCs, WJMSCs, ADMSCs, and BMMSCs by western blotting and ELISA, respectively. While the expression levels of MMP3 were similar in cell lysates from four different tissue-derived MSCs (Fig. 2c), the levels of secreted MMP3 were higher in TMSC-CM than in other MSC-CM (Fig. 2d). This finding was not statistically significant due to the donor-to-donor variation within the same MSC source. Therefore, further studies were conducted using the cells that secrete high levels of MMP3.

### MMP3 secreted from TMSCs promotes BMC engraftment

**Fig. 3:**
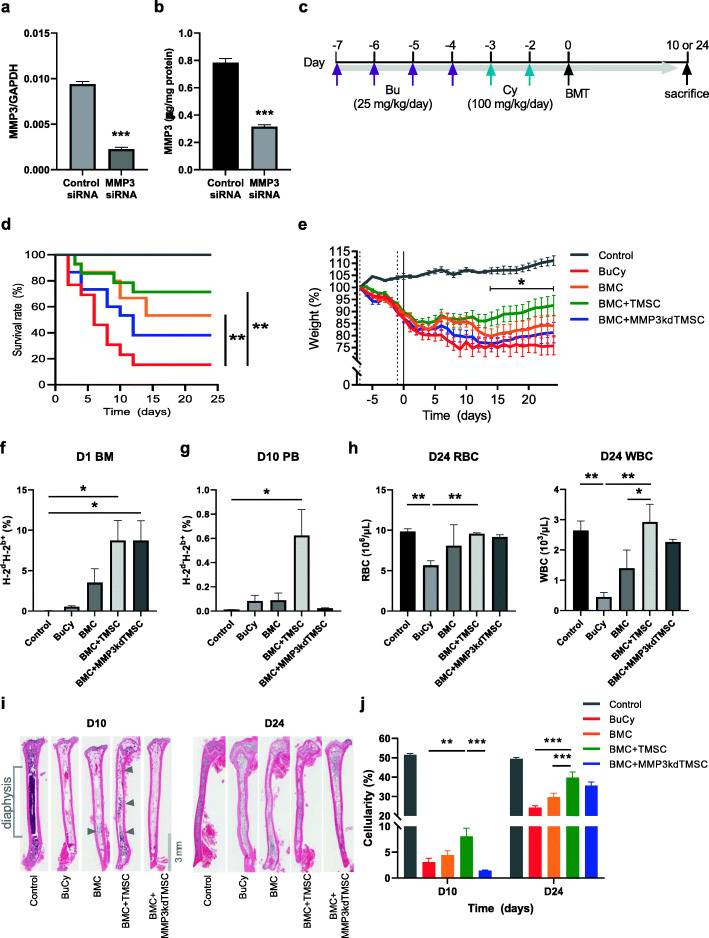
Survival, weight changes, and bone marrow engraftment after co-transplantation of BMCs and TMSCs. **a** The mRNA expression levels of MMP3 in control and MMP3-knockdown TMSCs. GAPDH was used as the reference gene. **b** MMP3 protein concentrations in control TMSC-CM and MMP3-knockdown TMSC-CM were quantified using a human MMP3 ELISA kit. **c** Experimental scheme: BALB/c mice were treated with the Bu/Cy regimen, transplanted after 48 h, and sacrificed 10 or 24 days after BMT. **d** Mouse survival was measured using Kaplan-Meier estimator and statistical analysis was performed using log-rank test (**P < 0.01). **e** Body weight changes from day − 7 to day 24 after transplantation. The data represent the mean ± S.E.M. and were analyzed using two-way ANOVA (*P < 0.05, compared with weights of the Bu/Cy group on the day specified). **f** Analyses of MHC haplotype expression in bone marrow cells on day 1 and **g** blood mononuclear cells on day 10 using flow cytometry. H-2^d^ for donor C57BL/6 and H-2^b^ for BALB/c recipient. **h** The numbers of RBCs and WBCs in peripheral blood were examined using Auto Hematology Analyzer. **i** The representative histology of the femur medullary cavity stained with H&E. Bone marrow sections were obtained from mice sacrificed on days 10 and 24. Reconstituted areas of the bone marrow are indicated by the gray arrows. Magnification × 20. **j** The bone marrow cellularity of the medullary cavity of the femur diaphysis was calculated. Data were analyzed using one-way ANOVA (*P < 0.05, **P < 0.01; ***P < 0.001)

In order to investigate the effects of MMP3-secreting TMSCs on BMT, we performed loss-of-function studies using siRNA transfection. Before transplantation, TMSCs were transfected with either control or MMP3 siRNA. As determined by RT-qPCR, the knockdown efficiency was about 75.8% (Fig. 3a). Secretion of MMP3 was also determined by ELISA, and MMP3 siRNA transfection reduced MMP3 secretion by 60% (Fig. 3b). Preconditioned BALB/c mice were transplanted either with BMCs alone (BMC) or in combination with control (BMC + TMSC) or MMP3-knockdown TMSCs (BMC + MMP3kdTMSC). The mice were then sacrificed after 10 or 24 days (Fig. 3c). When the weight of a mouse dropped to less than 75% of its starting weight (weight on day –7), the mouse was sacrificed, and the survival rate was determined (Fig. 3d). Results showed a significant increase in survival in BMC and BMC + TMSC transplanted groups compared to Bu/Cy group, but MMP3kdTMSC co-transplantation limited the mouse survival. BMC + TMSC group showed the highest survival rate among the experimental groups: 71% (10 of 14 mice survived, green line). However, the BMC + MMP3kdTMSC group showed a drop of survival rate to 38% (6 of 15 mice survived, blue line). When TMSCs were co-transplanted with BMCs, mice recovered their body weights faster than other experimental groups, and significant increase was achieved from day 14 onwards compared to Bu/Cy group (Fig. 3e). A short-term homing of donor cells (H-2^b^) to bone marrow was determined a day after BMT. The results demonstrated that TMSC co-transplantation enhances bone marrow homing regardless of MMP3 expression (Fig. 3f). On day 10, engraftment was determined by analyzing donor cell percentage in peripheral blood mononuclear cells. A significant increase in the percentage of donor-derived cells was detected only in BMC + TMSC group while MMP3kdTMSC co-transplantation did not show the same effect (Fig. 3g). Recovery in blood cell counts were determined on day 24. BMC + TMSC group showed increase in the numbers of RBC and WBC to the levels similar to the control group, while BMC + MMP3kdTMSC group did not show a significant change (Fig. 3h). Bone marrow cellularity of femur diaphysis was analyzed on day 10 and 24 to determine bone marrow reconstitution (Fig. 3i). TMSC co-transplantation significantly increased bone marrow cellularity from day 10, suggesting a promotion of bone marrow engraftment. These effects were not detected in BMC + MMP3kdTMSC group. By day 24, bone marrow cellularity recovered in every surviving mouse, but a significant increase was only observed in BMC + TMSC group (Fig. 3j). These results suggest that co-transplantation of TMSC with intact MMP3 expression enhances bone marrow engraftment and BMT efficacy.

### MMP3 secreted from TMSC promotes collagen IV degradation and BMC migration in vitro

**Fig. 4:**
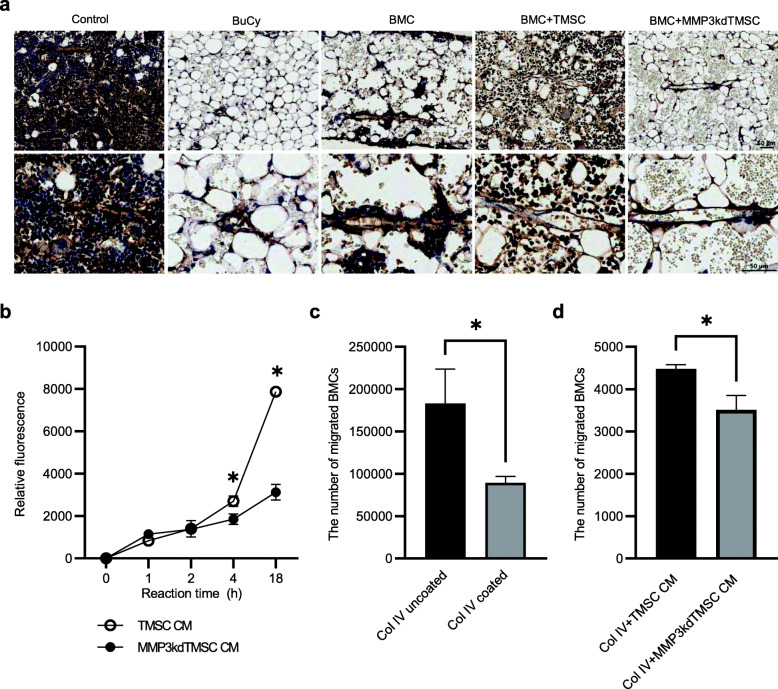
MMP3 secreted from TMSC promotes collagen IV degradation and BMC migration in vitro. **a** Immunohistochemical analyses of bone marrow basement membrane from mice sacrificed on day 10. Slides were stained with anti-collagen IV antibody and counterstained with hematoxylin. Representative images with a higher magnification focusing on bone marrow endothelium in lower panel. Scale bar 50 μm. **b** The enzymatic activity of MMP3 against collagen IV (Col IV) was measured using an EnzChek gelatinase/collagenase assay kit and fluorescently labeled DQ-type IV collagen. The x-axis represents the reaction time; the y-axis represents the relative fluorescence of degraded Col IV. The data represent the mean ± S.E.M. and were analyzed using two-way ANOVA (*P < 0.05). **c** A Transwell migration assay was conducted to confirm whether Col IV inhibited the migration of BMCs and **d** to confirm whether MMP3 accelerated the migration of BMCs in the presence of a Col IV barrier. Col IV was applied to the membrane inserts of a 24-well Transwell plate. BMCs were incubated with or without TMSC-CM for 4 h. The number of BMCs that migrated to the lower chamber was determined using 0.4% trypan blue staining. The data represent the mean ± S.E.M. and were analyzed using the Student’s t test (*P < 0.05)

Next, we examined histological changes of bone marrow basement membrane using collagen IV antibody on day 10. The results demonstrated thinned basement membrane lining the bone marrow endothelium in BMC + TMSC group compared to BMC or BMC + MMP3kdTMSC group (Fig. 4a). This supports our hypothesis that MMP3 expressing TMSC is involved in degradation of collagen IV. To confirm the modulation of collagen IV by MMP3-secreting TMSCs, a collagenase assay was performed. Degradation of collagen IV by treatment with TMSC-CM was observed while it is significantly decreased in MMP3-knockdown TMSC-CM treatment (Fig. 4b). To confirm whether MMP3 from TMSCs facilitate BMC chemotaxis in response to SDF-1 in the presence of a collagen IV barrier, we conducted a Transwell migration assay. BMC migration was hampered by 60% in the presence of collagen IV (Fig. 4c). In the presence of collagen IV and when supplemented with MMP3-knockdown TMSC-CM, the BMC migration rate was slower than control TMSC-CM (Fig. 4d), suggesting that MMP3 secreted from TMSCs promotes BMC migration by degrading the collagen IV barrier.

## Discussion

In this study, we showed that Bu/Cy preconditioning alters the ECM composition of the bone marrow, especially increases collagen IV expression. We hypothesize that injured ECM must recover to a normal state to facilitate BMC engraftment, and MSCs may play a role in this process. To investigate this idea, we screened the secreted proteins of TMSCs using a Mascot analysis and selected MMP3 as a candidate molecule. Using a mouse model of BMT, we co-transplanted TMSCs that were transfected with either control or MMP3 siRNA. The results demonstrated that co-transplantation of TMSCs enhanced mouse survival, bone marrow engraftment, and blood cell recovery; but these effects were not observed when MMP3 expression was downregulated in TMSCs. We also showed the effects of TMSC-CM on collagen IV degradation and BMC migration via the secretion of MMP3.

To identify the genes that are regulated by bone marrow preconditioning, we harvested mouse bone marrow after preconditioning and analyzed the changes in the expression of ECM genes using a pathway-targeted PCR array. Among the 30 significantly changed genes related to the ECM and adhesion molecules, 26 genes were upregulated in the preconditioned BM: *Col4a1* was the most highly upregulated gene and *Col4a2*, another constituent of collagen IV, was also upregulated. The most abundant ECM proteins in the bone marrow are fibronectin, collagens I–XI, laminin, tenascin, thrombospondin, and elastin [32]. Collagen IV is a network-forming molecule and major component of the basement membrane in bone marrow [33]. Because cell migration requires the degradation of the subendothelial basement membrane [34], we investigated whether TMSC co-transplantation would enhance BMC homing by modulating the ECM.

MMP3, part of the MMP family that participates in development, tissue repair, and pathological processes such as tumorigenesis and metastasis [35], was selected as a protein expressed in TMSCs that could modulate collagen IV based on the results of the Mascot analysis. Its expression was confirmed using RT-qPCR and an ELISA. MMPs, which are known to be expressed at low levels under normal conditions, exhibit increased expression during tissue remodeling, inflammation, and cancer progression. Human MMPs have similar structures but vary in their expression profiles and substrate specificities [36]. Target substrates include ECM molecules, other MMPs, proteinase inhibitors, growth factors, cytokines, and cell adhesion molecules [37]. MMP3 is known to degrade the basement membrane, especially collagen IV [38]. Using MMP3-knockdown TMSCs, the effects of MMP3 on survival and engraftment were determined.

The average body weights of mice that received the Bu/Cy combination regimen were significantly decreased on the day of transplantation (day 0) compared to their weights before the initiation of the regimen (day –7). When mice were co-transplanted with TMSCs, both survival and body weight were significantly increased; these effects were not observed with MMP3-knockdown TMSCs suggesting that MMP3 secreted from TMSCs play roles in enhancing BMT efficacy. Interestingly, MMP3 expression in TMSCs was not involved in short-term homing, as percentages of donor cells in the bone marrow were significantly increased in both BMC + TMSC and BMC + MMP3kdTMSC groups. However, MMP3kdTMSC failed to support BMC engraftment on day 10 and blood cell regeneration on day 24. These data demonstrate that MMP3 secreted from TMSCs is involved in the process of BMC engraftment at the bone marrow niches. Further studies may involve a more thorough investigation on which lineage differentiation of hematopoietic stem cells are modulated by TMSC co-transplantation.

The bone marrow cellularity of the BMC + TMSC group recovered to a greater extent than the other groups. According to previous reports, BMC engraftment can be assessed by histological methods [19, 39] and/or neutrophil and platelet counts [40]. In the present study, we examined BMC engraftment as the recovery of bone marrow cellularity. On day 10, bone marrow cellularity in the femur diaphysis was increased when TMSCs were co-transplanted with BMCs after the preconditioning regimen. On day 24, when the bone marrow reconstitution is almost completed in survived mice, BMC + TMSC group showed significantly increased cellularity compared to BuCy or BMC groups. These results are consistent with those of a previous report in which we showed that when BMCs were transplanted with TMSC-CM, bone marrow cellularity recovered faster than without TMSC-CM treatment [19]. In that study, we demonstrated that TMSCs recover injured bone marrow endothelial cells by the secretion of an angiogenic factor that promoted homing and engraftment of BMCs. The present work strengthens our previous findings and proposes another mechanism of action by which TMSCs enhance the efficacy of BMT.

Homing is a critical step in successful engraftment, which occurs within the first few hours up to 2 days after transplantation [41]. Some MMPs such as MMP2, MMP9, and MT1-MMP (MMP14) have been known to accelerate the homing of BMCs or MSCs by degrading the basement membrane and promoting cellular migration. When MMP2 and MT1-MMP were inhibited in human bone marrow MSCs, the invasive abilities of these cells were impaired in vitro [27]. MMP3 also facilitates the progression of cell migration and invasion by degrading ECM protein substrates such as collagen types II, IV, and IX, and activating other MMPs such as MMP1, 7, and 9 [28, 29]. Therefore, an increase in the rate of engraftment in the BMC + TMSC group compared to the BMC + MMP3kdTMSC group may be due to the ability of MMP3 to accelerate BMC homing by degrading the ECM, especially collagen IV.

In the current study, we demonstrated that TMSC co-transplantation enhances BMT efficacy. However, a development of MSC therapy for the application in HSCT can be challenging. One of the challenges is tracking the distribution of MSCs that are infused systemically. In vivo fluorescent imaging and/or quantification of human gene expression in various organs [42, 43] should be performed in preclinical level for securing safety of MSC therapy.

## Conclusion

Collectively, these results suggest that MMP3 expressed in TMSCs enhances BMC engraftment by degrading collagen IV in the bone marrow ECM and facilitating BMC migration. These results could be translated as a novel strategy for shortening the duration of pancytopenia in HSCT patients and enhancing HSCT efficacy.

## Data Availability

The data used to support the findings of this study are available from the corresponding author upon request.

## Abbreviations

ADMSC: Adipose tissue-derived mesenchymal stem cell
BMCs: Bone marrow cells
BMMSC: Bone marrow-derived mesenchymal stem cell
BMT: Bone marrow transplantation
Bu: Busulfan
CM: Conditioned medium
Col IV: Type IV collagen
Cy: Cyclophosphamide
ECM: Extracellular matrix
HSCs: Hematopoietic stem cells
HSCT: Hematopoietic stem cell transplantation
MMP: Matrix metalloproteinase
MSCs: Mesenchymal stem cells
SDF-1: α-Chemokine stromal-derived factor 1
TMSCs: Tonsil-derived mesenchymal stem cells
TMSC-CM: Conditioned medium of tonsil-derived mesenchymal stem cell
WJMSCs: Wharton’s jelly-derived mesenchymal stem cell
